# High Structural Stress and Presence of Intraluminal Thrombus Predict Abdominal Aortic Aneurysm ^18^F-FDG Uptake: Insights From Biomechanics

**DOI:** 10.1161/CIRCIMAGING.116.004656

**Published:** 2016-11-15

**Authors:** Yuan Huang, Zhongzhao Teng, Maysoon Elkhawad, Jason M. Tarkin, Nikhil Joshi, Jonathan R. Boyle, John R. Buscombe, Timothy D. Fryer, Yongxue Zhang, Ah Yeon Park, Ian B. Wilkinson, David E. Newby, Jonathan H. Gillard, James H. F. Rudd

**Affiliations:** From the Department of Radiology (Y.H., Z.T., Y.Z., J.H.G.), EPSRC Centre for Mathematical and Statistical Analysis of Multimodal Clinical Imaging (Y.H.), Department of Engineering (Z.T.), Division of Cardiovascular Medicine (M.E., J.M.T., I.B.W., J.H.F.R.), Wolfson Brain Imaging Centre (T.D.F.), and Statistical Laboratory (A.Y.P.), University of Cambridge, United Kingdom; British Heart Foundation Centre for Cardiovascular Science, University of Edinburgh, United Kingdom (N.J., D.E.N.); Department of Vascular Surgery (J.R. Boyle) and Department of Nuclear Medicine (J.R. Buscombe), Addenbrooke’s Hospital, Cambridge, United Kingdom; and Department of Vascular Surgery, Changhai Hospital, Shanghai, China (Y.Z.).

**Keywords:** abdominal aortic aneurysm, fluorodeoxyglucose F18, inflammation, mechanical stress, positron-emission tomography, thrombosis

## Abstract

**Background—:**

Abdominal aortic aneurysm (AAA) wall inflammation and mechanical structural stress may influence AAA expansion and lead to rupture. We hypothesized a positive correlation between structural stress and fluorine-18-labeled 2-deoxy-2-fluoro-d-glucose (^18^F-FDG) positron emission tomography–defined inflammation. We also explored the influence of computed tomography–derived aneurysm morphology and composition, including intraluminal thrombus, on both variables.

**Methods and Results—:**

Twenty-one patients (19 males) with AAAs below surgical threshold (AAA size was 4.10±0.54 cm) underwent ^18^F-FDG positron emission tomography and contrast-enhanced computed tomography imaging. Structural stresses were calculated using finite element analysis. The relationship between maximum aneurysm ^18^F-FDG standardized uptake value within aortic wall and wall structural stress, patient clinical characteristics, aneurysm morphology, and compositions was explored using a hierarchical linear mixed-effects model. On univariate analysis, local aneurysm diameter, thrombus burden, extent of calcification, and structural stress were all associated with ^18^F-FDG uptake (*P*<0.05). AAA structural stress correlated with ^18^F-FDG maximum standardized uptake value (slope estimate, 0.552; *P*<0.0001). Multivariate linear mixed-effects analysis revealed an important interaction between structural stress and intraluminal thrombus in relation to maximum standardized uptake value (fixed effect coefficient, 1.68 [SE, 0.10]; *P*<0.0001). Compared with other factors, structural stress was the best predictor of inflammation (receiver-operating characteristic curve area under the curve =0.59), with higher accuracy seen in regions with high thrombus burden (area under the curve =0.80). Regions with both high thrombus burden and high structural stress had higher ^18^F-FDG maximum standardized uptake value compared with regions with high thrombus burdens but low stress (median [interquartile range], 1.93 [1.60–2.14] versus 1.14 [0.90–1.53]; *P*<0.0001).

**Conclusions—:**

Increased aortic wall inflammation, demonstrated by ^18^F-FDG positron emission tomography, was observed in AAA regions with thick intraluminal thrombus subjected to high mechanical stress, suggesting a potential mechanistic link underlying aneurysm inflammation.

## Abstract 2

Supplemental Digital Content is available in the text.

Abdominal aortic aneurysm (AAA) is a localized enlargement of the aorta, conventionally diagnosed when the maximum anteroposterior vessel diameter exceeds 3.0 cm.^1^ AAAs are most prevalent in elderly men and are frequently asymptomatic until point of rupture, which is the catastrophic failure of the aneurysmal wall associated with an overall mortality rate between 65% and 85%.^2^ Current management is largely based on serial imaging to determine the maximum baseline diameter and rate of growth, with open or endovascular repair typically recommended for AAAs >5.5 cm. However, clinical trials and autopsy studies suggest that some small- and medium-sized AAAs will also rupture, whereas some large aneurysms can remain static for many years.^3^ Thus, aneurysm diameter alone cannot reliably identify high-risk AAAs, highlighting the need of better risk stratification.

**See Editorial by Curci and Beckman**

**See Clinical Perspective**

AAAs result from pathological changes occurring within the aortic wall that lead to tissue degradation and smooth muscle cell apoptosis; often adventitial and transmural inflammatory cell infiltrates are seen when examined histologically.^4,5^ These pathological changes are associated with AAA expansion and rupture,^6,7^ and inflammation in particular may cause extensive media damage.^8,9^ To quantify aortic wall inflammation in patients with aneurysm, fluorine-18-labeled 2-deoxy-2-fluoro-d-glucose (^18^F-FDG) positron emission tomography (PET) has been described.^10,11^ It enables detection of the increased glucose metabolic rate that is characteristic of aortic wall inflammation.^5^

Pulsatile loading because of changes in blood pressure and flow under physiological conditions also contribute to the pathogenesis of AAA, in part because of extracellular matrix breakdown.^12,13^ Ultimately, AAA rupture occurs when forces within the AAA structure exceed its inherent tissue strength. Biomechanical analysis can be applied to evaluate risks of AAA rupture^14,15^ by modeling structural stress, derived from several factors including aneurysm morphology and composition with excellent intra-/interobserver reproducibility.^16^ The overall relationship between structural stress and AAA inflammation has been studied by several groups.^17–19^ However, other aneurysm components, including intraluminal thrombus (ILT) and calcium, have only been partially considered in previous studies. In addition, the possible coinfluence of structural stress and aneurysm architectural features on ^18^F-FDG uptake remains unexplored. In this study, we aimed to quantify the distribution of structural stress within AAAs, modeling the effect of both calcification and ILT, and the association between local ^18^F-FDG uptake and structural stress, AAA morphology, and composition.

## Methods

### PET-CT Imaging

Patients with AAA were recruited from Cambridge University Hospitals, United Kingdom. The inclusion criteria were age >50 years and presence of an aneurysm between 3.0 and 5.5 cm. Exclusion criteria were insulin-dependent diabetes mellitus, type 2 diabetes mellitus with a fasting glucose of >11 mmol/L, any disease expected to shorten life expectancy to <2 years, women of childbearing age not taking contraception, severe renal failure (serum creatinine >250 µmol/L), known contrast allergy, or the inability to provide informed consent. All subjects provided written informed consent in accordance with the research protocol approved by the local institutional review board (MREC 09/H0308/27). All patients underwent ^18^F-FDG PET-computed tomography (CT) imaging of the aorta using a GE Discovery 690 PET/CT scanner (GE Healthcare). The details of imaging protocol are provided in Material in the Data Supplement.

### AAA Segmentation and Finite Element Simulation

**Figure 1.**
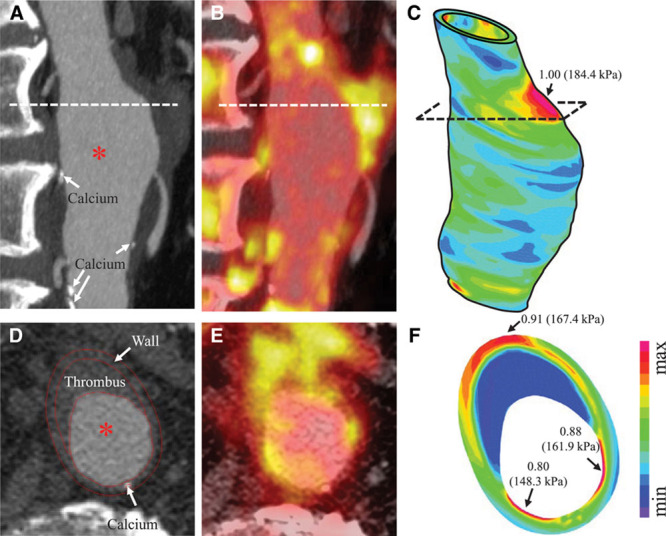
In vivo images and the calculated structural stress of an abdominal aortic aneurysm: (**A**) contrast-enhanced computed tomography (CT), sagittal view; (**B**) fluorine-18-labeled 2-deoxy-2-fluoro-d-glucose (^18^F-FDG) positron emission tomography (PET), sagittal view; (**C**) structural stress plotted on the 3-dimensional geometry (both normalized and absolute values); (**D**) contrast-enhanced CT, transverse view; (**E**) ^18^F-FDG PET, transverse view; and (**F**) structural stress plotted on the transverse plane (both normalized and absolute values).

AAA segmentation was performed using contrast-enhanced aortic CT images (Figure 1A and 1D). The luminal contour was delineated using a region-growing algorithm, and the outer wall was segmented manually. Because of the poor contrast between ILT and wall in CT image, the outer boundary of ILT was obtained by uniformly shrinking the wall contour inward with the assumption of constant wall thickness^17^ and the region enclosed by this shrunk wall contour and lumen boundary was defined as ILT (Figure 1D). Calcium was segmented using relative signal intensity,^20^ with different percentage threshold from 100% to 160% in steps of 5% with reference to the mean lumen signal intensity. The results were then compared with the manual delineation of 2 experienced clinicians, and the best agreement was found with the threshold of 110%.

Patient-specific finite element models were constructed (Figure 1C). The 2-dimensional slices were axially stacked together, and axial smoothing was performed to reconstruct the 3-dimensional geometry. A volume curve-fitting technique was used by dividing the 3-dimensional domain into hundreds of small volumes to curve-fit the irregular geometry with component inclusions.^21^ The entire structure was therefore meshed using hexahedron elements.

Because the configuration acquired in the CT images was pressurized, both circumferential and axial shrinkage was performed to determine the computational start shape.^22^ Patient-specific blood pressure was used as the loading condition. Material properties of aneurysmal tissues were provided in Material in the Data Supplement. Numeric models were solved using ADINA 8.7 (ADINA R&D Inc). The von Mises stress was used as the quantitative indicator of the biomechanical environment (Figure 1C and 1F), with its top 5 percentile values being discarded to minimize the influence of mesh imperfection. For each patient, the stress was normalized by its peak value within the AAA wall to provide the mechanics-defined relative severity in the range of 0 to 1. Although both ILT and calcium were considered when mechanical analyses were performed, only stress in the wall was extracted for analysis because aneurysm rupture is the result of wall failure.

### Image Coregistration and Octant-Based Data Sampling

The ^18^F-FDG PET and contrast-enhanced CT images were resampled and coregistered (Figure 1) using rigid transformation to minimize the signal intensity difference of the vertebra in a custom platform written in MATLAB R2014a (The MathWorks, Inc), allowing measurement of the maximum aortic wall standardized uptake value (SUV_max_). For precise matching of stress and PET data sets, each consecutive resampled axial slice throughout the length of the AAA (defined by max anteroposterior diameter >3.0 cm) was radially divided into 8 octants with area of 41.34 (30.07–72.46) mm^2^ (interquartile range). The AAA neck was defined as the segment of aorta bordering the first slice of aneurysmal aorta (≈6.5 mm above and below the first >3.0 cm diameter slice), and the sac comprises the region between the aneurysm neck and the inferior aspect of the aneurysm. In addition to stress and ^18^F-FDG SUV_max_ within the wall, ILT ratio and calcium ratio were also determined for each octant.

### Statistical Analysis

All statistical analysis were performed in MATLAB, with statistical significance assumed if *P*<0.05. As the data were nested (8 octants within each slice and multiple slices for each AAA), linear mixed-effects models were used to appropriately account for the hierarchical data structure (details provided in Material in the Data Supplement). Both uni- and multivariate analyses were performed. Backward elimination, which started with more complicated linear mixed-effects models and removed the insignificant effects based on the Akaike information criterion, was used in the multivariate analysis. Moreover, to better quantify the contribution of structural stress, multivariate models without (the null model) and with (the full model) the consideration of stress were obtained and compared.

The power of each fixed effect was evaluated using the receiver-operating characteristic curve, and Youden criterion was used to identify the optimal operating point. To determine whether 2 sets of data differ significantly from each other, 2-tailed Student *t* test or Mann–Whitney test was used where appropriate.

## Results

### Clinical Demographics, AAA Morphology, and Composition

In total, 9192 octants were included in the analysis from 21 patients. Mean age was 78.4±6.7 years, and 19 of the 21 (90.5%) subjects were male. Six of the 21 patients (28.6%) had previous cardiovascular disease. Mean AAA size was 4.10±0.54 cm. More patient demographics and descriptions of each aneurysm can be found in Table 1.

**Table 1.**
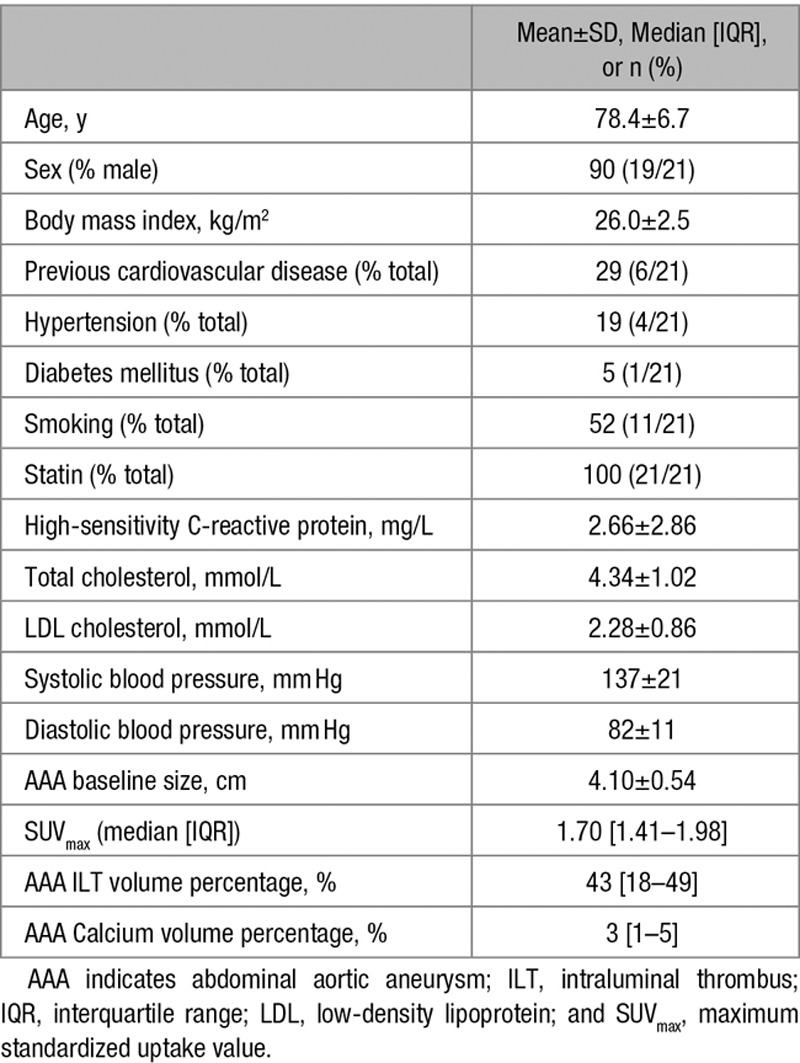
Characteristics of the Abdominal Aortic Aneurysm Subjects (n=21)

**Figure 2.**
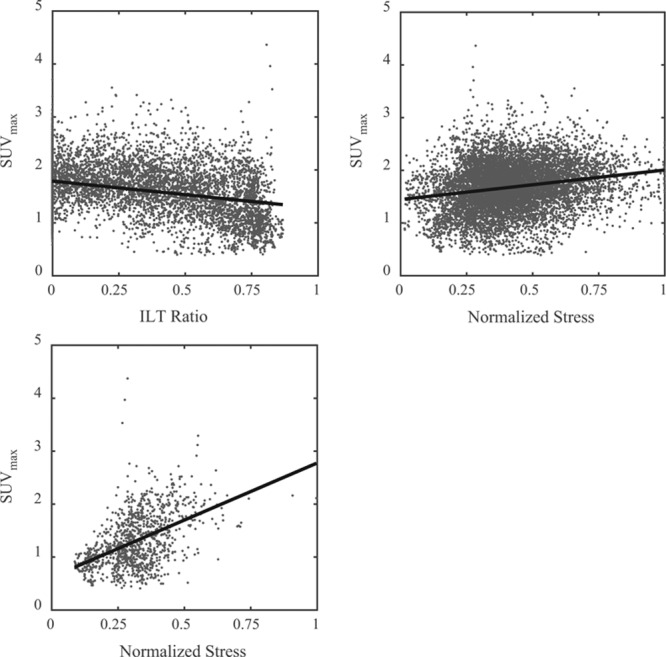
Scatter plot between the maximum standard uptake value (SUV_max_) of fluorine-18-labeled 2-deoxy-2-fluoro-d-glucose positron emission tomography and morphological features or structural stress: (**A**) SUV_max_ vs intraluminal thrombus (ILT) ratio; (**B**) SUV_max_ vs normalized stress in all regions; and (**C**) SUV_max_ vs normalized stress in regions with ILT ratio >0.67.

**Figure 3.**
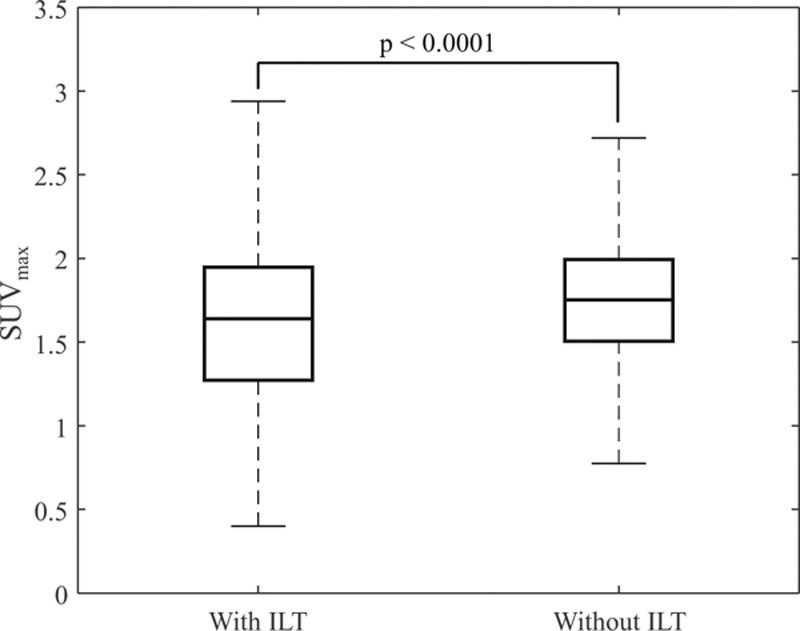
Comparison of maximum standardized uptake value (SUV_max_) in regions with and without local intraluminal thrombus (ILT).

Using the linear mixed-effects model, when results from all octants were pooled, slice outer wall diameter, ILT ratio, and calcium ratio were all found to be statistically significant predictors of ^18^F-FDG uptake; in particular, SUV_max_ decreased with increasing ILT ratio (Figure 2A); whereas luminal diameter and patient demographic factors, including age, sex, body mass index, diabetes mellitus, and blood pressure, were not predictive (Table 2). To identify the hierarchical importance of these effective predictors, a multivariate linear mixed-effects analysis was performed (Table 3). Although statistically significant (*P*<0.0001), both local maximum wall diameter and calcium ratio had only a small effect on prediction of aortic SUV_max_. ILT ratio had a larger effect on SUV_max_, with each 10% increase in ILT area decreasing the SUV_max_ by 0.121 (*P*<0.0001) in the wall. This was confirmed by comparing the SUV_max_ in octants with and without ILT. Octants with thrombus demonstrated a lower SUV_max_ than the ILT-free octants (number of octants [number of patients]: 4475 (n=21) versus 4717 (n=21), SUV_max_: 1.64 [1.27–1.95] versus 1.75 [1.51–1.99], *P*<0.0001; Figure 3). However, octants with ILT were associated with a larger SUV_max_ range than those without (with ILT: 0.40–2.94 versus without ILT: 0.77–2.72; Figure 3).

**Table 2.**
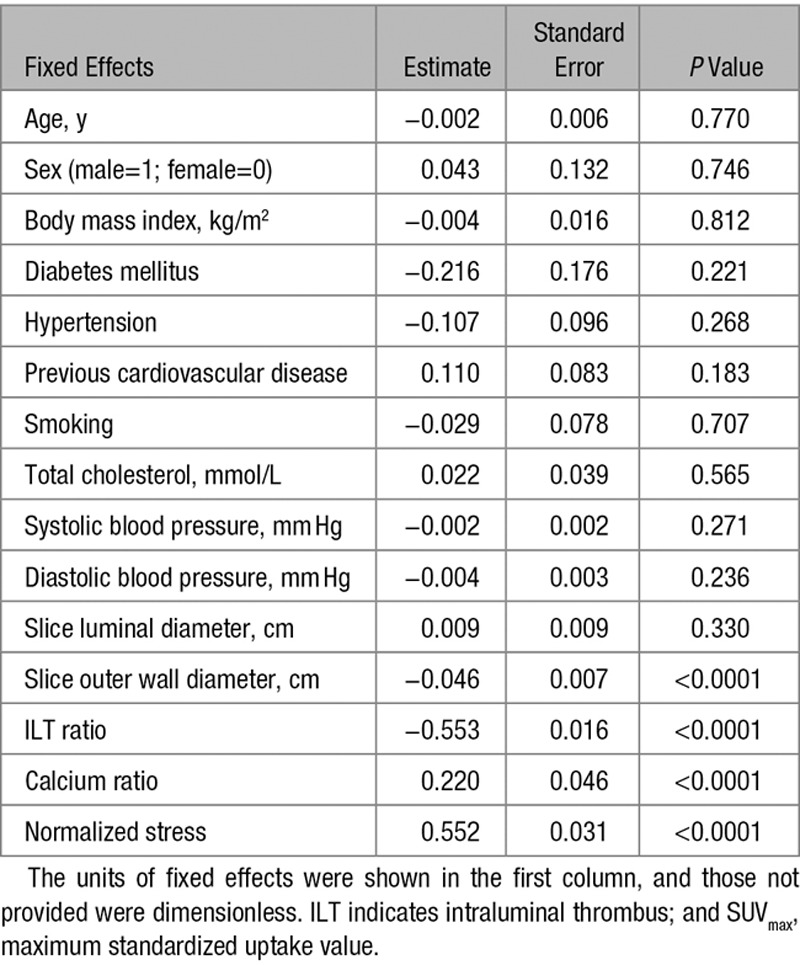
Fixed Effects Coefficients of the Univariate Linear Mixed Effects Analysis With Respect to SUVmax

**Table 3.**
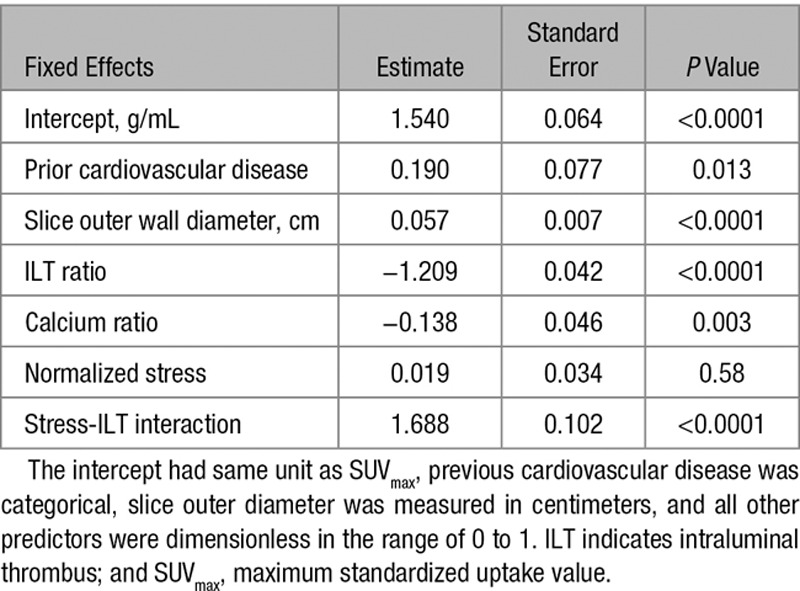
Fixed Effects Coefficients of the Full Linear Mixed Effects Model of SUVmax

The random effects covariance parameters (psi) associated with octant number, anatomic location, and patient subjects are 0.012, 3.464×10^−4^, and 0.025, respectively. This suggests, for instance, a standard deviation of 0.158 (95% confidence level [0.116–0.215]) in SUV_max_ associated with the difference among individual patients. Such random effects arise from the hierarchical data structure that should be properly considered using mixed-effects models.

### Structural Stress Versus ^18^F-FDG Uptake

**Figure 4.**
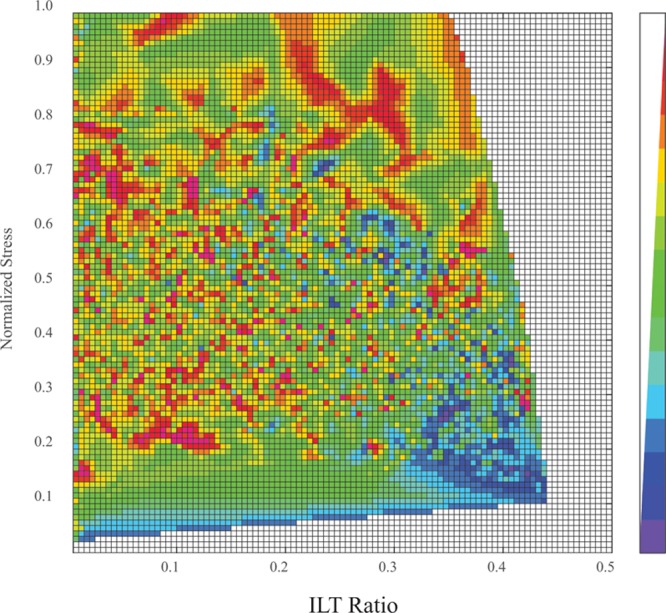
Heat map showing the relation between maximum standardized uptake value (SUV_max_), intraluminal thrombus (ILT) ratio, and normalized stress. The value of SUV_max_ is indicated by the color of each grid.

When comparing normalized stress and SUV_max_ in all wall regions using the univariate linear mixed-effects model, a positive correlation was observed with a slope estimate of 0.552 (*P*<0.0001; Table 2; Figure 2B). An enhanced relationship was observed in octants with ILT occupying >67% of the area (Figure 2C). This impact of ILT content was confirmed by multivariate linear mixed-effects analysis. In the multivariate model, although normalized stress itself did not demonstrate any statistical significance in the prediction of SUV_max_, the interaction between normalized stress and ILT ratio leads to an increase in SUV_max_ (*P*<0.0001; Table 3 and Figure 4).

### The Incremental Value of Structural Stress in Defining High ^18^F-FDG Uptake

Multivariate linear mixed-effects analysis was performed both with and without the inclusion of stress. Compared with the model without considering stress, the full linear mixed-effects model lead to a reduction of Akaike information criterion value by 344.3. This indicated that stress was an important effect and should not be neglected. Therefore, results reported for the multivariate analysis were based on the model with stress.

**Figure 5.**
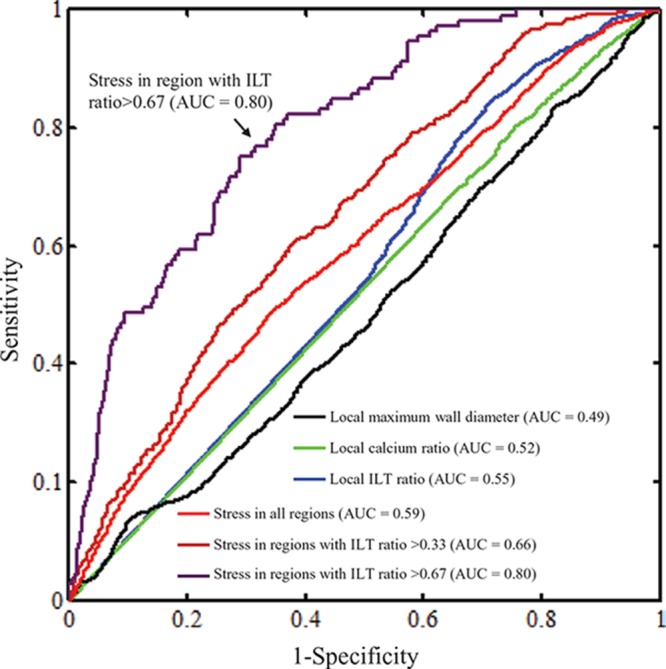
Serial receiver-operating characteristic curve analyses comparing the capability of differentiating regions with high (maximum standardized uptake value [SUV_max_]>2) and low (SUV_max_≤2) fluorine-18-labeled 2-deoxy-2-fluoro-d-glucose uptake. The structural stress lead to an improved prediction compared with abdominal aortic aneurysm morphology and composition, and this further improved with the combination of the extent of local intraluminal thrombus (ILT). AUC indicates area under the curve.

**Figure 6.**
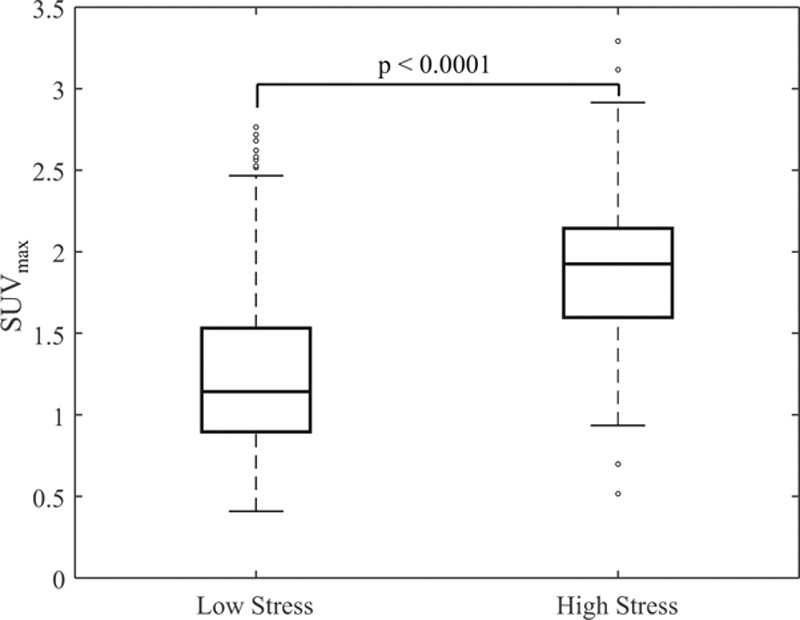
Comparison of maximum standardized uptake value (SUV_max_) in regions with low (≤0.45) and high (>0.45) structural stress. This comparison was performed in the regions with intraluminal thrombus (ILT) ratio >0.67, and the stress threshold was identified earlier in the receiver-operating characteristic curve analyses.

Receiver-operating characteristic curve analysis was performed to identify factors that might help predict octants with high (SUV_max_>2) and low (SUV_max_≤2) ^18^F-FDG uptake^23^ and to determine the optimal threshold for stress in relation to ^18^F-FDG uptake. Normalized stress (AUC=0.59; Area Under the Curve) was a better predictor of ^18^F-FDG uptake when compared with morphological features, including maximum outer diameter (AUC=0.49), ILT ratio (AUC=0.55), and calcium ratio (AUC=0.52; Figure 5). AUC of stress increased from 0.59 to 0.66 when limited to octants with ILT ratio >0.33 (n=2894 from 20 patients). For octants with ILT ratio >0.67 (n=1064 from 16 patients), the AUC of stress further rose to 0.80. In this subset, the optimal operating point for the differentiation of SUV_max_ was identified as a normalized stress of 0.45. When using a 0.45 stress threshold to determine high and low stress among 1064 octants with ILT ratio >0.67, higher ^18^F-FDG uptake was found in the high stress group (median [interquartile range]: 1.93 [1.60–2.14] versus 1.14 [0.90–1.53]; *P*<0.0001; Figure 6).

## Discussion

In this study, we explored relationships between wall structural stress, AAA morphology and compositions, and inflammation determined by ^18^F-FDG PET. In the case of aneurysm wall, there is reasonable evidence that FDG preferentially accumulates in inflamed areas.^5,10,24^ We observed a link between stress and inflammation, particularly in aneurysmal regions with high ILT. To the best of our knowledge, this is the first retrospective clinical study to quantify the effect of AAA morphology and composition with regard to wall stress and inflammation.

**Figure 7.**
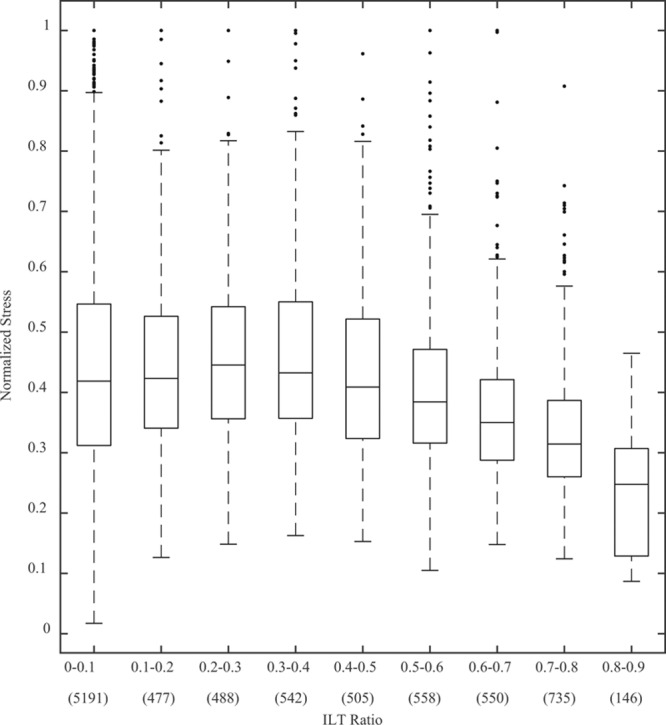
Relation between normalized stress and local intraluminal thrombus (ILT) ratio within each octant (the figure in the brackets blow the *x* axis showed the number of octants).

ILT plays a pivotal role in the pathogenesis of AAA, with both potentially protective and deleterious effects. Although ILT seems in part to protect against AAA rupture through a cushioning effect that reduces wall stress,^25,26^ it also weakens the vessel wall, increasing rate of AAA expansion.^27^ Our findings mirror this dichotomy. Overall, there was a negative correlation between ILT ratio and SUV_max_ (Figure 2A), which is possibly explained by lower wall stress because of increased ILT area (Figure 7). We observed increased ^18^F-FDG uptake in relation to harsh mechanical conditions in the aortic wall beneath a thick ILT. This finding is supported by previous studies showing increased inflammatory infiltration in AAA wall beneath ILT.^28^ Heavy ILT burden might also lead to local hypoxia and neovascularization,^29^ which could, in principle, augment cellular ^18^F-FDG uptake.^30^ A causal relationship between high structural stress and AAA inflammation has previously been suggested.^12^ At the cellular level, among other pathogenic mechanisms, high stress increases reactive oxygen species production within vascular smooth muscle cells, which activates nuclear factor-kappa B^31^ and promotes inflammation.^32^ Mechanical loading might also cause enhanced proteolytic activities in macrophages.^33^

Aneurysm is a multicomponent inhomogeneous structure with irregular geometry. Its components and geometric determinants undoubtedly influence the mechanical environment within the structure^25,34^ (Figure I in the Data Supplement). If calcium and ILT were treated as wall, the stress distribution changed dramatically (Figure II in the Data Supplement). The multivariate analysis in this study showed that compared with local luminal and outer wall diameter, both percentage ILT and calcium had a greater influence in stress calculation. Although in general, stress within the structure decreased as ILT increased (Figure 6), as shown in Figure IA in the Data Supplement, high stress areas are still found in regions with a high ILT ratio. This study also showed that in addition to ILT, calcium ratio was associated with ^18^F-FDG uptake negatively although the effect was weak (Table 3). It may be because of the ability of calcium in undertaking mechanical loading increases with its size. As a result, stress in AAA wall decreased while calcium ratio increases (Figure IB in the Data Supplement).

In this study, modeled scatter correction is applied by the GE Discovery 690 PET/CT scanner, which should limit the degrading impact of scatter. The scanner uses a relatively narrow energy window (425–650 keV) to limit scattered coincidences, in combination with modeled scatter correction as part of the image reconstruction algorithm that includes compensation for multiple scattered events and single scatter events. The partial volume effect resulting from limited spatial resolution, however, is a limitation of this study that may affect FDG value used for analysis; ^18^F-FDG PET signals relating to the aneurysm wall might reflect some spillover from nearby structures, such as ILT, in addition to true aortic wall uptake. To assess the partial volume effect on the results of the present study, we increased the wall thickness inward by 1 pixel (≈0.6 mm), re-extracted the SUV_max_ within the wall and compared with our original results. SUV_max_ in these 2 scenarios remained highly consistent; the difference was <1% when comparing all octants (original wall thickness: 1.70 [1.41–1.98] versus increased wall thickness 1.71 [1.42–1.98]) and <2% when comparing octants with ILT ratio >0.67 (1.20 [0.93–1.65] versus 1.22 [0.94–1.66]). We therefore concluded that partial volume effect was associated with little impact onto the quantitative results in this study. Such an observation could be explained by the spatial distribution of inflammatory infiltration in the ILT. Previous studies using immunohistochemical analysis^35,36^ and flow cytometry^37^ have shown that the extent of inflammatory cell infiltration decreases significantly with increasing distance to lumen, for example, the average percentage of macrophages measured 8 mm from the vessel lumen reduced to ≈3%, compared with significantly higher percentage at the luminal surface.^35^ Therefore, although signal spillover from ILT might contribute to ^18^F-FDG AAA wall signal intensity, it is likely that the highest inflammatory signals originating from ILT occur close to the lumen where they are less likely to significantly influence the signal within AAA wall providing ILT is thick.

One of the key findings in the present study is that the combination of stress and local ILT burden, rather than either factor alone, could lead to better prediction of increased ^18^F-FDG uptake in AAA wall. The interaction term in the linear mixed effect model suggests that the role of mechanical stress becomes increasingly important with greater ILT ratio, which is also shown in the receiver-operating characteristic curve analysis where AUC improved when the analysis was limited to regions with thick ILT. Mechanics showed relatively poorer predictive power in regions with no or thin thrombus, which could potentially be explained by several factors: (1) further morphological stratification of these regions may be needed to differentiate, for example, an octant with little ILT formation versus an octant from AAA with heavy ILT burden arising at the so-called shoulder area that lacks thrombus but where inflammation remains high; and (2) in octants with less ILT, the partial volume effect might be more likely to influence apparent ^18^F-FDG uptake within the wall than in octants with thick ILT as described above. The main clinical benefit from our findings would be the ability to identify a subgroup of patients with small AAAs but high FDG uptake, ILT load, and mechanical stress. These aneurysms may experience more rapid expansion than average, requiring more frequent surveillance and prophylactic repair before rupture. However, further longitudinal studies, with clinical outcomes, are warranted to determine the clinical significance of these findings.

Overall, our results are consistent with previous studies comparing finite element analysis-derived structural stress and ^18^F-FDG PET. In one study, Xu et al^17^ compared the ^18^F-FDG uptake and structural stress in 5 patients with aneurysms in the descending thoracic or abdominal aorta. Using a visual comparison, authors reported that high structural stress regions colocalized with areas of elevated ^18^F-FDG uptake. Furthermore, both high structural stress and increased ^18^F-FDG uptake occurred at the site of future rupture in 2 cases. In another study, Maier et al^18^ showed a positive correlation (*r*=0.71; *P*=0.0005) between ^18^F-FDG uptake and structural stress, including 18 patients with AAA. Moreover, the ^18^F-FDG uptake was resampled and mapped to the finite element geometry and significant element-wise correlation between structural stress and ^18^F-FDG uptake was seen in 16 of 18 AAAs. In the largest study to date, Nchimi et al^19^ investigated 53 patients with either infrarenal or thoracic aortic aneurysms, and a modest correlation between AAA stress and inflammation quantified by ^18^F-FDG uptake was found using multivariate linear regression (*r*=0.26; *P*=0.0009). Our study not only confirmed these findings but also showed the potential of using structural stress, in combination with AAA composition, to differentiate the low/high ^18^F-FDG uptake regions.

The present study has several limitations and assumptions. First, although each patient underwent a contrast-enhanced CT aortogram, inherent limitations of CT imaging sometimes made delineation of the aortic outer wall boundary difficult. Despite our best efforts to accurately delineate the structural components during the segmentation process, it was not always possible to define the exact border between AAA wall and ILT. We therefore assumed a uniform wall thickness for our calculations. In reality, AAA wall thickness will vary depending on multiple factors,^38^ such as ILT burden^28^ and spatial location.^39,40^ A sensitivity analysis with models of both uniform and variable wall thickness was performed. Stresses calculated from both models showed excellent correlation (Figures II and III in the Data Supplement). Therefore, we are confident that the limitations of our border delineation did not have a material effect on overall results. Second, limited CT tissue contrast also precluded the use of an anatomic-based PET partial volume correction in our analysis. This may be possible as PET/magnetic resonance imaging becomes more available. Third, no side branches were considered for stress modeling. Fourth, piece-wise homogeneous, isotropic material was used for the simulations, and the influence of fiber orientation was not accounted for. Finally, the computational models were structure-only, and effects because of blood flow were not considered. However, 3-dimensional structure-only analysis is computationally inexpensive and can provide accurate results.^41^

## Conclusion

Increased inflammation depicted by ^18^F-FDG was observed in AAA wall regions subjected to high mechanical stresses, especially in the presence of significant ILT. Because of the complicated 3-dimensional structure of AAA, the cushioning effect of ILT might be ineffective in certain circumstances, resulting in a combination of high stress and weakened aortic wall in the presence of ILT that might accelerate inflammation and other pathogenic processes in AAA. The present study provides a mechanistic insight into the relationship between structural stress and inflammation.

## Acknowledgments

We thank Dr Shahin Tavakoli and Mr Tengyao Wang from the Statistical Laboratory, Department of Pure Mathematics and Mathematical Statistics, University of Cambridge, for the assistance in statistical analyses.

## Sources of Funding

This study was supported by the British Heart Foundation Cambridge Centre of Excellence (RE/13/6/30180), Heart Research UK (RG2638/14/16), EPSRC Centre for Mathematical and Statistical Analysis of Multimodal Clinical Imaging (EP/N014588/1), and the National Institute for Health Research (NIHR) Cambridge Biomedical Research Centre. Dr Tarkin is supported by a Wellcome Trust research training fellowship (104492/Z/14/Z). Dr Rudd is part-supported by the NIHR Cambridge Biomedical Research Centre, the British Heart Foundation, the Wellcome Trust, and Higher Education Funding Council for England (HEFCE). Dr Newby is supported by the British Heart Foundation (CH/09/002) and is the recipient of a Wellcome Trust Senior Investigator Award (WT103782AIA).

## Disclosures

None.

## Supplementary Material

## CLINICAL PERSPECTIVE

Inflammation burden in the aortic abdominal aneurysm structure has been shown to be associated with patient clinical presentations, subsequent lesion progression, and deadly rupture. This study quantified the association between local ^18^F-FDG uptake and structural stress in aortic abdominal aneurysm, lesion morphology, and compositions, including intraluminal thrombus and calcium. The coinfluence of structural stress and aneurysm architectural features on ^18^F-FDG uptake-defined wall inflammation was demonstrated, suggesting a novel mechanistic link underlying aneurysm inflammation. This finding might enable the ability to identify a subgroup of patients with small aortic abdominal aneurysms but high FDG uptake, intraluminal thrombus load, and mechanical stress. These aneurysms might experience more aggressive disease development than average, requiring more frequent surveillance and prophylactic repair before rupture. Further longitudinal studies, with clinical outcomes, are warranted to determine the clinical significance of this finding.
