# Supplementary material for: High Structural Stress and Presence of Intraluminal Thrombus Predict Abdominal Aortic Aneurysm 18F-FDG Uptake: Insights From Biomechanics
Source: Circ Cardiovasc Imaging. 2016 Nov 15;9(11):e004656. doi: 10.1161/CIRCIMAGING.116.004656 (PMC5113243; doi:10.1161/CIRCIMAGING.116.004656)
Supplement: Supplementary file 1 [file hci-9-e004656-s001.pdf]

Supplemental Material

Supplement to: Huang Y, Teng Z, Elkhawad M et al. High Structural Stress and Presence of Intraluminal Thrombus Predict Abdominal Aortic Aneurysm ^{18}F-FDG Uptake: Insights from Biomechanics

PET-CT Imaging Protocol

Patients fasted for at least 6 hours prior to imaging, consistent with established vascular PET imaging protocols[1]. A target activity of 240 MBq ^{18}F-FDG was injected intravenously after which patients rested in a quiet environment for 90 minutes before being transferred onto the PET/CT scanner. A low dose CT was performed for attenuation correction, followed by a PET scan covering three bed positions from the arch of aorta to the aortic bifurcation over 30 minutes (10 minutes per bed position). Tracer circulation times were based on previous studies using ^{18}F-FDG in atherosclerosis[2], aimed to produce optimal contrast between the aortic wall and the blood pool. With the patient lying in the same position, a CT aortogram from the diaphragm to the aortic bifurcation was performed using 75-100 mL of iodinated contrast (400 mgI/mL; Iomeron, Bracco, Milan, Italy), followed by 50 mL of 0.9% saline flush. PET data were reconstructed using the time of flight ordered subsets expectation-maximization (TOF-OSEM) algorithm implemented on the scanner, which incorporated the following data corrections: dead time, random coincidences, normalization, scatter, attenuation, sensitivity and radioactive decay.

Material properties of aneurysmal tissues

Tissues, including wall, ILT and calcium, were assumed to be hyperelastic, homogeneous, isotropic and incompressible with material properties described by the modified Mooney-Rivlin formulation:

$$W = c_1(\bar{I}_1 - 3) + D_1\left[exp\left(D_2(\bar{I}_1 - 3)\right) - 1\right] + \kappa(J - 1),$$

where $\bar{I}_1 = J^{-2/3}I_1$ and $J = det(\boldsymbol{F})$, $\boldsymbol{F}$ is the deformation gradient and I_1 is the first invariant of deformation tensor. κ is Lagrangian multiplier for the incompressibility. c_1, D_1, and D_2 are material parameters derived from previous experimental studies: arterial wall, c_1=0.07 kPa,

D_1=6.54 kPa, D_2=5.88; ILT, c_1=0.24 kPa, D_1=8.69 kPa, D_2=0.61; and calcium, c_1=7.24x10^3 kPa, D_1=0.01 kPa, D_2=2.34x10^{-14} [3-5].

Construction of linear mixed-effect models

Fixed-effect predictors included in the statistical model were: (I) clinical demographics and risk factors (age, gender, body-mass index, diabetes mellitus, blood pressure, prior cardiovascular events and smoking); (II) morphological measurements (slice luminal diameter, slice outer wall diameter, ILT ratio and calcium ratio); and (III) structural stress. Octant number, anatomical location (neck or sac) and patient subject were modeled as the random effects.

Compositional and geometrical features effecting the stress in the AAA wall

The deformation of each aneurysmal component was governed by the Cauchy momentum equation,

$$\rho_s \ddot{\boldsymbol{U}} = \nabla \cdot \boldsymbol{\sigma}$$

where $\boldsymbol{U}$ is the displacement vector, $\boldsymbol{\sigma}$ is the stress tensor and ρ_s is the density of each component. In the case of big deformation, the governing equation is non-linear. Moreover, the material property of each component is non-linear[3] and the geometry of each component is irregular. These factors interact with each other non-linearly, effecting the mechanical condition within the aneurysm structure. As shown in Figure S1 below, local luminal, outer wall diameter, ILT and calcium all significantly affected stress in the aneurysm, but the latter two had greater effects. It is worthwhile pointing out that although in general stress decreases when ILT increases (Figure S1A), the stress varies widely in regions with a large ILT ratio.

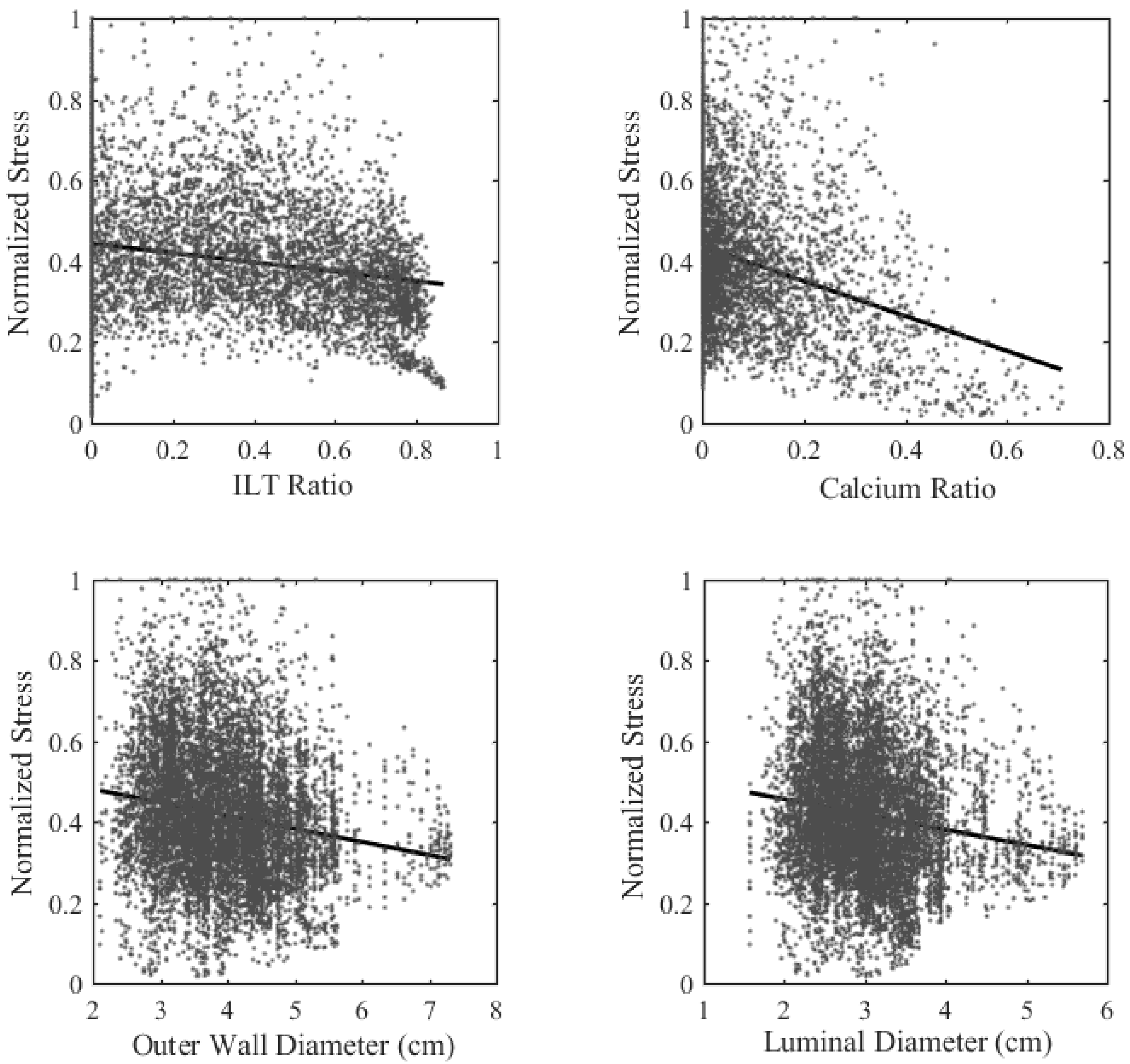

Figure S1. Association between stress and aneurysmal compositional and geometric features (A: Normalized stress vs ILT ratio; B: Normalized stress vs calcium ratio; C: Normalized stress vs outer wall diameter; and D: Normalized stress vs luminal diameter)

If the calcium and ILT were treated as wall, the stress distribution changed dramatically. The high stress concentration beneath the thick ILT layer, observed in Figure S2A and D, disappeared due to this over-simplification. Similar observations were seen in this study, where the stress level in ILT was low due to it being a softer material[6] and calcium undertook high stress loading due to its stiffer nature[4]. In order to accurately calculate the stress distribution within AAA wall, both ILT and calcium should be considered.

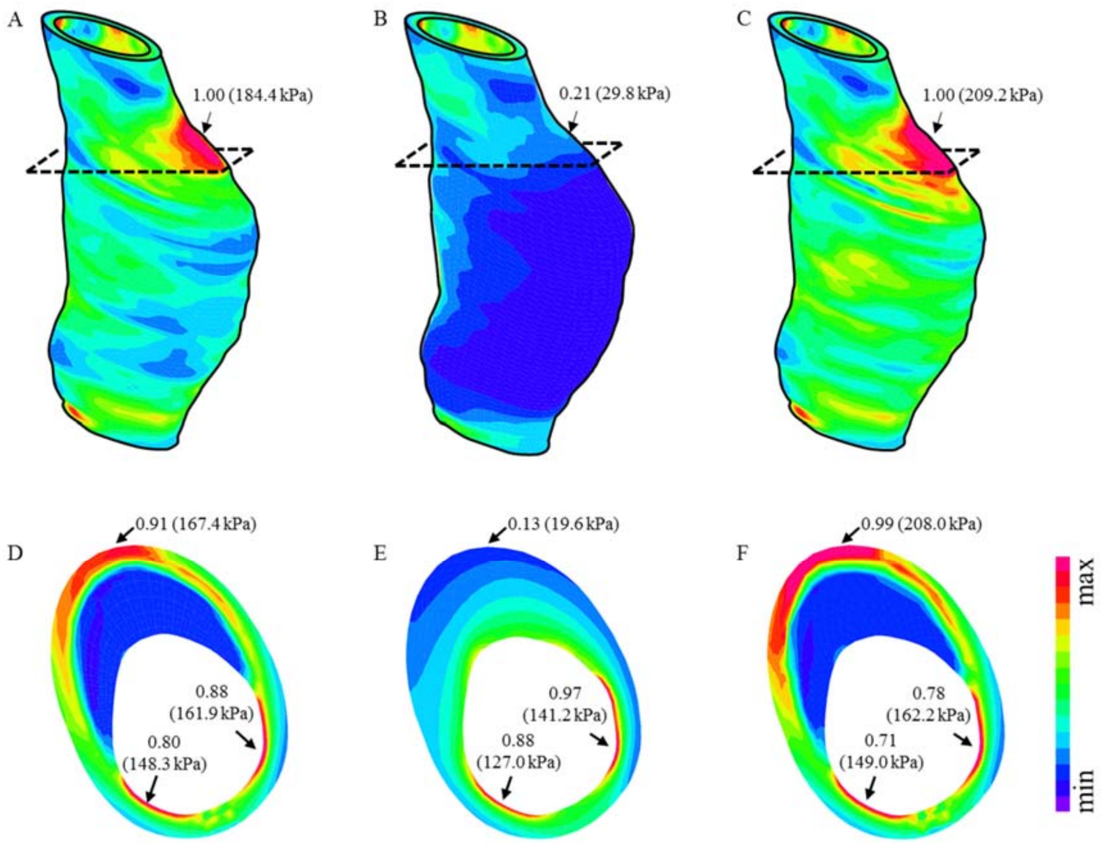

Figure S2. Stress distributions with different modeling assumptions. Upper panel: band plot on 3D geometry: (A) the model with uniform wall thickness and inclusion of components; (B) the model with uniform wall thickness but treating ILT and calcium as wall; and (C) the model with variable wall thickness and inclusion of components. Lower panel (D-F) shows the corresponding band plot on the transverse plane.

To better understand the influence of variations in wall thickness, a sensitivity analysis with models of both uniform and variable wall thickness (UWT and VWT, respectively) was performed. Compared with the VWT simulation, the stresses calculated in the UWT model showed excellent correlation (Figure S2C and F) with only a slight underestimation (slope=1.008, intercept=-5.5kPa, Pearson r=0.94, p<0.0001, Figure S3).

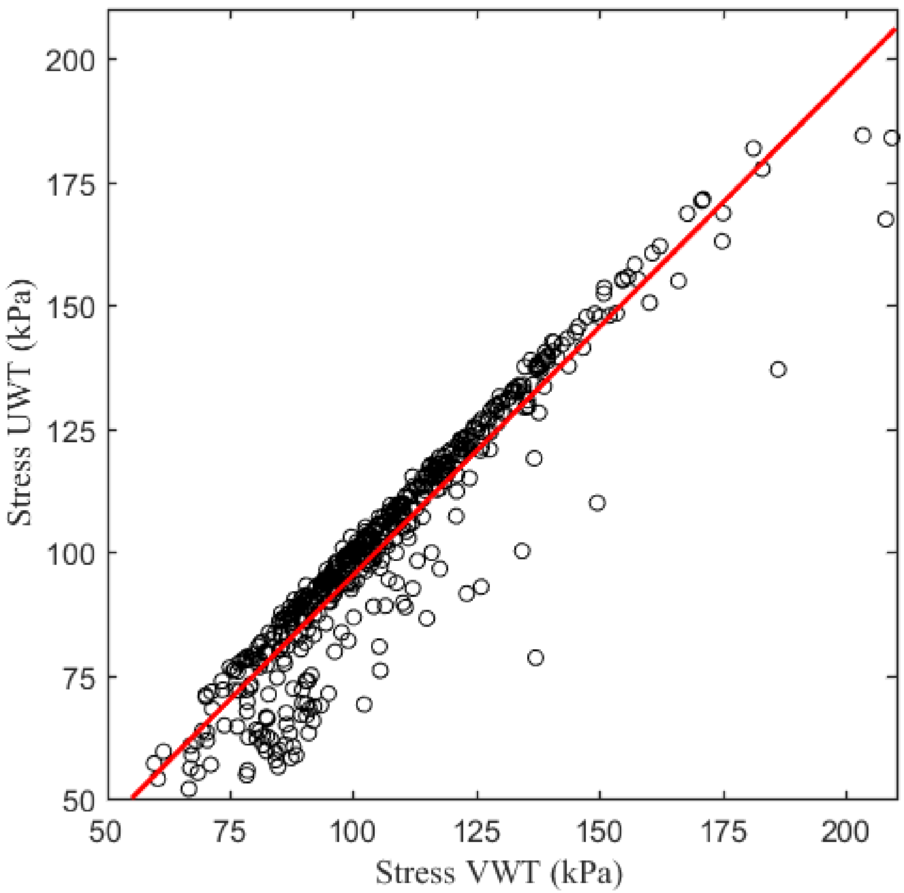

Figure S3. Correlation between stress values based on the assumption of uniform (UWT) and variable wall thickness (VWT).

Supplemental References

1. Maki-Petaja KM, Elkhawad M, Cheriyan J, Joshi FR, Ostor AJ, Hall FC, Rudd JH and Wilkinson IB. Anti-tumor necrosis factor-alpha therapy reduces aortic inflammation and stiffness in patients with rheumatoid arthritis. *Circulation*. 2012;126:2473-80.
2. Rudd JH, Warburton EA, Fryer TD, Jones HA, Clark JC, Antoun N, Johnstrom P, Davenport AP, Kirkpatrick PJ, Arch BN, Pickard JD and Weissberg PL. Imaging atherosclerotic plaque inflammation with [18F]-fluorodeoxyglucose positron emission tomography. *Circulation*. 2002;105:2708-11.
3. Teng Z, Feng J, Zhang Y, Huang Y, Sutcliffe MP, Brown AJ, Jing Z, Gillard JH and Lu Q. Layer- and Direction-Specific Material Properties, Extreme Extensibility and Ultimate Material Strength of Human Abdominal Aorta and Aneurysm: A Uniaxial Extension Study. *Ann Biomed Eng*. 2015;43:2745-59.
4. Maier A, Gee MW, Reeps C, Eckstein HH and Wall WA. Impact of calcifications on patient-specific wall stress analysis of abdominal aortic aneurysms. *Biomechanics and modeling in mechanobiology*. 2010;9:511-21.
5. Ebenstein DM, Coughlin D, Chapman J, Li C and Pruitt LA. Nanomechanical properties of calcification, fibrous tissue, and hematoma from atherosclerotic plaques. *Journal of biomedical materials research Part A*. 2009;91:1028-37.
6. Wang DH, Makaroun MS, Webster MW and Vorp DA. Effect of intraluminal thrombus on wall stress in patient-specific models of abdominal aortic aneurysm. *Journal of vascular surgery*. 2002;36:598-604.
